# Onset of subcutaneous emphysema and pneumomediastinum after tonsillectomy: a case report

**DOI:** 10.1016/S1808-8694(15)31292-1

**Published:** 2015-10-20

**Authors:** Ângelo C.D. Panerari, Ana C. Soter, Flavio La Porta da Silva, Luis F. de Oliveira, Mayra D'Andrea R. Neves, Antonio C. Cedin

**Affiliations:** 1 Resident physician, Clínica de Otorrinolaringologia Ivan F. Barbosa, Hospital Beneficência Portuguesa de São Paulo; 2 Coordinator of Medical Residence in Otorhinolaryngology, Clínica Ivan F. Barbosa, Hospital Beneficência Portuguesa de São Paulo

**Keywords:** pneumomediastinum, subcutaneous emphysema, tonsillectomy

## Abstract

Several complications can be related to surgical approaches of head and neck regions. Among those, there are rare conditions such as pneumomediastinum, pneumothorax and subcutaneous cervical emphysema. This study reports a case of a patient that developed pneumomediastinum, pneumothorax and subcutaneous emphysema after undergoing tonsillectomy. In order to reduce these complications in surgical approaches such as tonsillectomy, care should be taken with intubation, use of oxygen mask for positive pressure ventilation during anesthesia recovery, aggressive surgical maneuvers and use of surgical instruments that may cause deep tonsillar injuries.

## INTRODUCTION

Several complications may be associated with surgical procedures in the head and neck regions. According to Chen, among those are pneumomediastinum, pneumothorax and subcutaneous emphysema of face and neck. Subcutaneous emphysema following facial trauma, tooth extraction, tonsillectomy and sinusal surgery has been described by Friedman et al. The reported case presented pneumomediastinum, pneumothorax and subcutaneous emphysema after being submitted to palatine tonsillectomy. The purpose of the present study was to discuss mechanisms, morbidity, types of prevention and treatment of these conditions.

## CASE REPORT

A 31-year-old female patient – I.S.F. - was submitted to palatine tonsillectomy with diagnosis of chronic angina. She went on surgery under general anesthesia with orotracheal intubation. Initially, incision of the anterior pillar was done with scalpel 12, 3mm from the edge, inferior and upward 0.5cm from side of uvula. Dissection of palatine tonsil was performed on the subcapsular plane to avoid excessive trauma. Hemostasis was performed by means of bipolar electrocoagulation. The surgical procedure progressed uneventfully.

Immediately after extubation, the patient started to recover from anesthesia with oxygen mask; however, swallow and crepitating were verified bilaterally on the anterior region of neck with progressive extension to submentonian, mentonian, facial and anterior thoracic regions.

Patient was reintubated and submitted to surgical revision. Air bubbles in the lower region of the right tonsil were observed after local administration of small quantity of sterile solution. A chrome 3-0 catgut thread was used to suture and close muscular planes.

**Figure 1:**
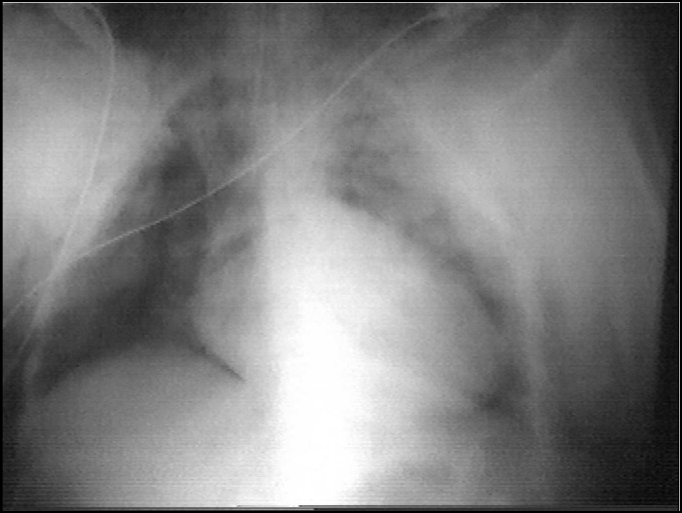
Immediate postoperative obse7rvation showing pneumomediastinum.

As patient was extubated, the swollen extended to anterior thorax, neck and mentalis region. Breathing stridor and dyspnea were observed. Radiological exploration of the anterior-upper region of neck and thorax revealed pneumomediastinum and extensive subcutaneous emphysema around the anterior region of thorax, neck and mentalis. Pharynx and trachea did not present any abnormalities (Figure 1).

A tracheostomy was performed to unblock the airways. Gatifloxacin and clindamicin were administered immediately after surgery and maintained up to the 10^th^ day.

**Figure 2:**
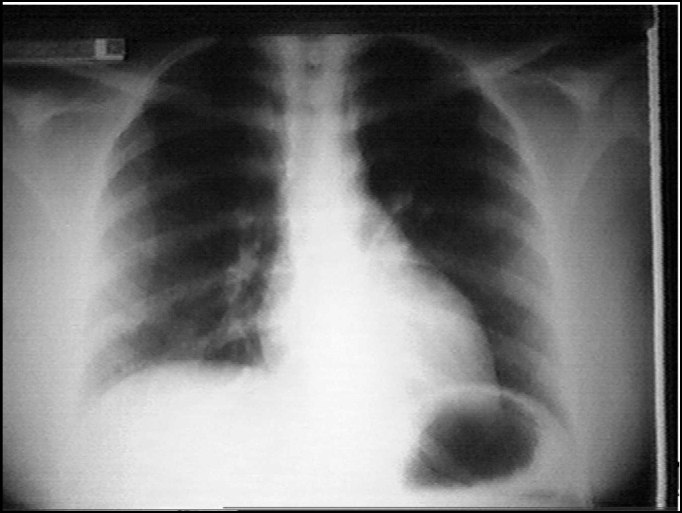
5^th^ Postoperative visit showing radiological resolution of pneumomediastinum.

Progressive reduction of subcutaneous emphysema followed by full disease regression at end of the 5^th^ day of surgery were achieved. Patient was decannulated as breathing was normal when tracheostoma was occluded; radiological exploration revealed solved pneumomediastinum (Figure 2).

## DISCUSSION

Differential diagnosis of neck and face swollen after palatine tonsillectomy includes hematoma, cellulitis, allergic reaction, angioedema, subcutaneous emphysema and pneumomediastinum^1^.

Laennec firstly described pneumomediastinum as a traumatic complication in 1819^2^. In general, it is considered a benign and self-limited condition, not requiring surgical intervention, although etiology must be elucidated to exclude baseline diseases.

Most frequently, the causes of pneumomediastinum are: secondary condition to increased intrathoracic pressure, Valsalva maneuver, extenuating exercising, weight lifting, vomiting, use of cocaine and other use of nasal drugs, barotrauma and asthma. Uncommon causes of pneumomediastinum include: arthroscopy, tooth extraction, palatine tonsillectomy, trombone playing, maneuvers that require maximum exhaling resistance and endotracheal intubation with pyriform sinus or vallecula lacerations^2^, ^3^.

In the reported case, an aspirator for tonsil dissection was used and patient was ventilated with positive oxygen pressure to return from anesthesia.

According to the literature, onset of iatrogenic subcutaneous emphysema of face and neck in oral cavity surgical approaches may occur due to aggressive technique, inappropriate equipment with high air compression drills, inadequate central venous approach and post-palatine tonsillectomy, when patient is submitted to ventilation with positive oxygen pressure for anesthetic recovery^4^, ^5^, ^6^, ^7^.

Head and neck subcutaneous emphysema produce variable manifestations, from discrete discomfort and crepitating at tissues palpation to involvement of aerodigestive ways leading to pain, severe swollen and crepitating, in case of penetration of a large volume of air, as observed in the present case. Moreover, it is important to highlight that orbital emphysema may cause visual impairment.

Treatment of subcutaneous emphysema varies according to level of severity and to surgeons' expertise. Most cases of subcutaneous emphysema are solved after two or three days of supportive treatment and residual swollen is usually minimal after 7-10 days of observation.

Surgical approach for decompression of extensive emphysema is not routinely adopted, once apparently it is ineffective, not mentioning the possibility of disease aggravation.

Treatment should be conservative in the majority of cases and based on the nature and benign course of subcutaneous emphysema. Moreover, in case of recurrent emphysema or respiratory disorders, precaution and preventive measures should be taken.

In severe cases with airways involvement, orotracheal intubation and tracheotomy are indicated, as occurred in the present case.

## CLOSING REMARKS

As to prevent emphysematous complications from palatine tonsil surgeries, intubation should be carefully performed to avoid upper airway lesions. Use of oxygen mask for positive pressure ventilation should be avoided during patients' anesthetic recovery, especially if there is significant tissue laceration due to dissection.

Ultimately, the surgical approach should be the least aggressive, avoiding vigorous maneuvers and use of inadequate instruments that increase the possibility of unnecessary traumas to tonsils.
